# Dying to pay: end-of-life medical costs for middle-aged and older adult patients with cardiovascular and cerebrovascular diseases

**DOI:** 10.3389/fpubh.2025.1548999

**Published:** 2025-03-12

**Authors:** Guoheng Hu, Haining Zhao, Xiaolong Bian, Ying Li

**Affiliations:** Yanshan University, Qinhuangdao, Hebei, China

**Keywords:** cardiovascular and cerebrovascular diseases, end-of-life healthcare expenditures, terminal stage, propensity score matching (PSM), difference-in-differences machine learning (DDML)

## Abstract

**Objective:**

This study aims to investigate end-of-life healthcare expenditures among middle-aged and older patients with cardiovascular and cerebrovascular diseases, with a particular focus on the existence of the “nearing-death effect.”

**Methods:**

Using inpatient discharge summary data from the Chinese National Medical Insurance Settlement Platform, we identified a cohort of middle-aged and older adults (aged 45 and above) diagnosed with cardiovascular and cerebrovascular diseases in Province H, China, during 2018–2019. Propensity Score Matching (PSM) was employed to examine differences in end-of-life healthcare expenditures between deceased and surviving patients. Robustness checks were performed using Multidimensional Fixed Effects (MDFE) and Difference-in-Differences Machine Learning (DDML).

**Results:**

The findings reveal a substantial increase in end-of-life healthcare expenditures among patients with cardiovascular and cerebrovascular diseases. Specifically, Total Medical Costs, Comprehensive Service Fees, Diagnosis Fees, Treatment Fees, Pharmaceutical Fees, and Nursing Care Fees rose by 34.3, 44.0, 35.7, 62.5, 49.9, and 46.8%, respectively, all statistically significant at the 1% level. These results highlight a pronounced escalation in healthcare expenditures associated with patient mortality.

**Conclusion:**

Among middle-aged and older patients with cardiovascular and cerebrovascular diseases, healthcare expenditures exhibit a distinct “end-of-life effect,” characterised by a sharp surge in medical spending during the final stages of life. This phenomenon underscores the intensive utilization of medical resources at the end of life, markedly differing from healthcare expenditure patterns at other stages of life.

## 1 Introduction

The rising cost of healthcare, particularly at the end of life, has become a major challenge faced by healthcare systems worldwide. Research indicates that the proportion of total healthcare expenditure attributed to end-of-life care varies across countries, typically ranging from 10 to 13% (1–3). Cardiovascular and cerebrovascular diseases, among the leading causes of death globally, place a significant strain on healthcare resource consumption due to their high recurrence rates and fatality. In 2020, the total number of patients discharged from hospitals in China due to cardiovascular and cerebrovascular diseases reached 24.28 million, with associated medical expenses amounting to 270.9 billion yuan. These substantial medical costs not only exacerbate the financial burden on individuals and families but also raise concerns about inefficiencies in resource allocation and disparities in healthcare accessibility.

In this context, scholars have increasingly focused on the multifaceted determinants of end-of-life healthcare expenditure. Existing research indicates that the expenditure on end-of-life care is not solely determined by a single linear factor but rather emerge from the complex interplay of multiple factors, including patient characteristics, medical intervention models, decision-makers’ preferences, and the institutional environment. At the patient level, in particular, end-of-life healthcare costs exhibit significant temporal heterogeneity. The cost escalation in the final 3 months of life follows an exponential trajectory, primarily due to interventions for acute complications and the intensive utilization of critical care resources (4). Furthermore, age-related differences influence indirectly expenditure patterns through treatment choices, as younger patients, who are more likely to undergo aggressive treatments, typically incur higher end-of-life costs than older individuals.

The intensity of medical interventions is a key driver of rising factors healthcare costs, particularly at the end of life. In some cases, these interventions extend beyond medically beneficial treatments, leading to futile care, which underscores the imbalance between medical advancements and ethical considerations. A study by Schouel et al. (5) found that the per capita cost of life-sustaining treatments for patients with irreversible diseases in the ICU can be as high as $658,000, without yielding significant improvement in clinical outcomes. Such decisions are often heavily influenced by physicians’ practice preferences. Cutler et al. (6) noted that approximately 35% of end-of-life healthcare expenditure is attributed to treatment plans that are not aligned with evidence-based medicine.

Additionally, patients’ and their families’ demand for life-prolonging treatment are deeply shaped by cultural values. In certain religiously conservative regions, the heavy reliance on advanced medical technologies and the tendency to prolong treatment are particularly pronounced, creating a vicious cycle of “moral obligation-resource consumption” (7).

The institutional environment also plays a critical role in shaping the structure of healthcare costs. The social insurance system faces sustainability challenges due to the financial strain of an aging population, while ethical debates on cost-effectiveness assessments further complicate institutional decision-making (8). Transforming care models presents a viable path for cost optimization. Although the initial cost of home-based end-of-life care is relatively high, it can generate long-term savings by reducing readmission rates (9). Additionally, in regions where euthanasia is legal, the demand for high-intensity treatments has declined (10), further underscoring the significant impact of legal frameworks on healthcare cost structures.

Although existing literature has examined the driving mechanisms of end-of-life healthcare costs from multiple perspectives, two main research gaps remain. First, empirical validation of the “near-death effect” remains insufficient. Most studies rely on scenario analyses or simple cohort comparisons, making it difficult to disentangle the confounding effects of age, comorbidities, and time to death (11). Moreover, many studies treat the “near-death effect” as a given, lacking rigorous quantitative validation within a counterfactual framework. Second, research on disease specificity and institutional contexts remains limited. Current research predominantly focuses on diseases such as cancer, neglecting acute-onset conditions like cardiovascular and cerebrovascular diseases. Additionally, most studies are based on Western healthcare systems, with limited consideration of the payment environment structures under social health insurance in developing countries like China, which institutional factors may further intensify end-of-life cost pressures.

This study seeks to address these gaps by focusing on the middle-aged and older adult patients with cardiovascular and cerebrovascular diseases. Using inpatient medical record data, we apply the Propensity Score Matching (PSM) method to examine differences in end-of-life healthcare expenditures and the dynamic evolution of cost structures between deceased and non-deceased patients, thereby empirically validating the “near-death effect.” Additionally, we employ Multidimensional Fixed Effects (MDFE) and Difference-in-Differences Machine Learning (DDML) for robustness checks, effectively accounting for time heterogeneity and unobservable confounders.

This not only uncovers the distinct patterns of end-of-life healthcare expenditures but also carries significant practical implications for curbing the rapid escalation of medical costs. By providing empirical evidence, this study offers scientific support for policymakers in formulating cost-control strategies, thereby optimizing medical resources allocation, enhancing end-of-life care services, alleviating the financial burden on patients’ families, and promoting the sustainable development of healthcare systems.

## 2 Methods

### 2.1 Data source

The data used in this study were authorized by the Medical Insurance Settlement Platform of Province H in China. All datasets underwent anonymization and de-identification procedures to ensure that no personally identifiable information was disclosed in this article or its Supplementary materials. As administrative data, their usage strictly complied with provincial regulatory and legal frameworks governing administrative data applications. Given that this study constitutes a secondary analysis of anonymized administrative records, it does not involve human participants or experimental procedures requiring ethics review board approval. Furthermore, all methodological approaches adhered to the journal’s guidelines and prescribed standards.

A stratified random sampling approach was employed for data selection. First, urban stratification was conducted: the data provider categorized provincial hospitalization records into distinct strata based on municipal jurisdictions, with each stratum encompassing all records from its respective city. Second, systematic sampling was applied within each municipal stratum: hospitalization records were chronologically ordered by discharge month, and systematic sampling was performed at fixed intervals within each monthly subset. The systematically sampled records from all municipal strata were then aggregated, yielding a final sample of 111,819 cases.

In alignment with the research objectives, the analysis was restricted to patients aged 45 years or older with a diagnosis of cardiovascular or cerebrovascular diseases, yielding a final sample size of 44,341 cases. Case identification for these conditions was based on the first four digits of ICD-10 codes, with detailed specifications provided in Supplementary Table 1. Additionally, to mitigate the influence of extreme values on the model, all expenditure variables were winsorized at the 1% level.

H Province, as one of the fastest-aging regions in China, is highly representative due to its demographic and healthcare system characteristics aligning closely with the national average. According to the Seventh National Census, the proportion of older adult individuals in H Province (44.02%) is nearly identical to the national average (44.16%), ensuring that the sample reflects the broader aging trend in China. Additionally, the province’s basic medical insurance reimbursement ratio is consistent with national policies, and its government-led healthcare financing structure—where 67% of funding comes from the government—resembles the nationwide public health financing framework. These similarities indicate that the findings from H Province can provide valuable insights into the cost dynamics of end-of-life care across China. Moreover, given that H Province’s healthcare financing structure is also comparable to certain middle- and high-income economies, such as Turkey and Mexico, this study contributes to the global discourse on optimizing end-of-life healthcare policies within publicly funded healthcare systems.

### 2.2 Indicator selection

This study selects variables based on existing research, China’s healthcare context, and data availability. The dependent variable is healthcare expenditures for middle-aged and older patients with cardiovascular and cerebrovascular diseases, classified into six categories: Total Medical Costs, Comprehensive Service Fees, Diagnosis Fees, Treatment Fees, Pharmaceutical Fees, and Nursing Care Fees. Total Medical Costs refers to the sum of all medical expenses incurred by the patient during hospitalization. Following Ta et al. (12) and Zang et al. (13), the classification of medical expenses is as follows: Comprehensive Service Fees comprise general medical service fees, general treatment operation fees, nursing fees, rehabilitation fees; Diagnosis Fees cover pathology diagnosis fees, laboratory examination fees, radiological examination fees, clinical diagnosis item fees, and disposable medical material costs for examinations; Treatment Fees encompass non-surgical treatment item fees, surgical treatment fees, and disposable medical material fees for treatments or surgeries; Pharmaceutical Fees cover the costs of western medicine, Chinese herbal medicine, and traditional Chinese medicine preparations; Nursing Care Fees include basic nursing fees, graded nursing fees, consumables fees, and special service charges.

The key independent variable is patient mortality status, identified from discharge diagnosis. Control variables include age, occupation, marital status, method of medical payment, hospitalizations frequency, and length of hospital stay.

Descriptive statistics for the key variables are presented in Table 1, while disease-specific statistics are provided in Supplementary Tables 2, 3. The descriptive analysis reveals significant differences between the deceased and non-deceased groups across several variables. In terms of healthcare expenditure trends, the average total healthcare costs for the deceased group are significantly higher than those for the non-deceased group. Specifically, the deceased group exhibits a marked increase in spending on treatment fees, pharmaceutical fees, and nursing care fees. Overall, the analysis indicates considerable differences in both the level and structure of healthcare expenditures between the deceased and non-deceased groups, underscoring the substantial impact of mortality on the distribution and composition of healthcare costs.

**Table 1:** Descriptive statistical analysis.

Variable name	Full sample		Death group	Non-death group	
	Mean	Standard deviation	Mean	Mean	MeanDiff
Total medical costs	10.263	0.956	10.243	11.017	−0.775***
Comprehensive service fees	8.237	1.095	8.212	9.161	−0.949***
Diagnosis fees	8.303	1.368	8.284	9.009	−0.724***
Treatment fees	6.734	3.588	6.698	8.078	−1.380***
Pharmaceutical fees	8.765	1.176	8.737	9.802	−1.065***
Nursing care fees	6.176	1.818	6.145	7.345	−1.200***
Death	0.026	0.159	0	1	−1
Age	66.423	10.895	66.242	73.17	−6.927***
Occupation	0.077	0.19	0.077	0.077	0.000***
Payment method	0.111	0.235	0.111	0.111	0.003***
Marriage	0.2	0.202	0.19	0.175	−0.002***
Hospitalizations frequency	2.261	5.072	2.245	2.845	−0.600***
Length of hospitalization	33.05	22.51	32.62	49.099	−16.478***
Type 2 diabetes mellitus	0.191	0.393	0.191	0.184	0.007
Hyperlipidemia	0.072	0.258	0.073	0.014	0.060***
Fatty liver disease	0.032	0.176	0.033	0.005	0.028***
*N*	44,341	44,341	43,185	1,156	1,156

The values in parentheses represent *t*-values; ***, **, and * denote significance at the 1, 5, and 10% levels, respectively. The same applies to the following tables.

### 2.3 Model construction

This study employs Propensity Score Matching (PSM) to analyze the differences in healthcare expenditures between deceased and non-deceased middle-aged and older patients with cardiovascular and cerebrovascular diseases. By matching deceased patients with non-deceased patients sharing similar multidimensional characteristics, the model estimates the net impact of mortality on end-of-life healthcare expenditures, effectively addressing potential bias caused by sample imbalances. The model is structured as follows:

First, calculate the determinant equation for mortality among middle-aged and older patients with cardiovascular and cerebrovascular diseases Equation (1):


(1) $$\mathrm{ps}\left(X\right)=\mathrm{Pr}\left[D=1|X\right]=E\left[D|X\right]$$

Here, *X* represents the multidimensional factors influencing mortality among middle-aged and older patients with cardiovascular and cerebrovascular diseases, including Age, Occupation, Payment Method, Marriage, Number of Hospitalizations, Length of Hospitalization. *D* indicates whether the patient is deceased (1 = deceased, 0 = surviving). *ps* denotes the probability of mortality, also referred to as the propensity score.

Secondly, calculate the propensity score for each individual sample to estimate the average effect of patient mortality on end-of-life healthcare expenditures (Average Effect of Treatment on the Treated, ATT). The corresponding equation is as follows Equation (2):


(2) $$\begin{array}{c} ATT=E\left[Y_{1,i}-Y_{0,i}|D_{i}=1\right]\\ =E\left\{E\left[Y_{1,i}-Y_{0,i}\;|\;D_{i}=1,\;\mathrm{ps}\left(X_{i}\right)\right]\right\}\\ =E\left\{E\left[Y_{1i}\;|\;D_{i}=1,\;\mathrm{ps}\left(X_{i}\right)\right]-E\left[Y_{0i}\;|\;D_{i}=0,\;\mathrm{ps}\left(X_{i}\right)\right]|D_{i}=1\right\} \end{array}$$

Here, *Y_1i_* and *Y_0i_* represent the potential levels of end-of-life healthcare expenditures for deceased and surviving patients, respectively. The propensity score (*ps*) is estimated using a logit model,


(3) $$\mathrm{ps}\left(X_{i}\right)=\mathrm{Pr}\left(D_{i}=1|X_{i}\right)=\exp\left(\beta x_{i}\right)/\left(1+\exp\left(\beta x_{i}\right)\right)$$

As shown in Equation (3). Here, $X_{i}$ represents the multidimensional factors influencing patient mortality, including Age, Occupation, Payment Method, Marriage, Number of Hospitalizations, Length of Hospitalization. β denotes the coefficients of these multidimensional variables, while the *ps* value represents the predicted propensity score obtained from the logit model. Subsequently, this study employs nearest-neighbor matching, radius matching, and kernel matching methods to select paired samples.

Finally, to further investigate the differences in end-of-life healthcare expenditures between deceased and surviving middle-aged and older patients with cardiovascular and cerebrovascular diseases, the estimation model is specified as follows:


(4) $$y_{\mathrm{it}}=\alpha_{0}+\alpha_{1}X_{i}+\alpha_{2}X_{\mathrm{it}}+\alpha_{2}Z_{i}+\varepsilon_{\mathrm{it}}$$

As shown in Equation (4). Here, *i* and *t* represent the individual patient and the survey year, respectively. $y_{\mathrm{it}}$ denotes the dependent variable, which is end-of-life healthcare expenditures. *X* represents a set of observable control variables, including Age, Occupation, Payment Method, Marriage, Number of Hospitalizations, Length of Hospitalization. $\varepsilon_{\mathrm{it}}$ is the error term. This study primarily focuses on estimating the coefficients of the control variables for deceased patients, specifically Age, Occupation, Payment Method, Marriage, Number of Hospitalizations, Length of Hospitalization.

## 3 Results

Nearest Neighbor Matching is employed in Propensity Score Matching (PSM) to estimate the Average Treatment Effect on the Treated (ATT). This method selects the non-deceased individual with the closest propensity score to each deceased individual, ensuring an balance in the distribution of control variables between the deceased and non-deceased groups. This approach minimizes potential confounding bias and enhances the reliability of the causal effect estimation. The results are presented in Table 2.

**Table 2:** ATT values before and after nearest neighbor matching.

Variable	Sample	Treated	Controls	Difference	S.E.	T-stat
Total medical costs	Unmatched	11.027	10.268	0.759	0.028	27.22***
	ATT	11.027	10.658	0.369	0.033	11.04***
Comprehensive service fees	Unmatched	9.161	8.212	0.949	0.032	29.24***
	ATT	9.161	8.735	0.426	0.041	10.48***
Diagnosis fees	Unmatched	9.031	8.359	0.672	0.035	19.18***
	ATT	9.031	8.608	0.423	0.048	8.72***
Treatment fees	Unmatched	8.121	6.794	1.327	0.105	12.58***
	ATT	8.121	7.324	0.797	0.130	6.14***
Pharmaceutical fees	Unmatched	9.812	8.763	1.049	0.034	30.71***
	ATT	9.812	9.246	0.567	0.039	14.56***
Nursing care fees	Unmatched	7.402	6.282	1.121	0.047	23.71***
	ATT	7.402	6.912	0.490	0.058	8.44***

Table 2 reports the ATT values estimated using the nearest-neighbor matching method. A significantly different ATT from zero indicates a significant treatment effect.

The findings reveal that the end-of-life healthcare expenditures of deceased middle-aged and older patients with cardiovascular and cerebrovascular diseases differ significantly from zero at the 5% level. This suggests a substantial disparity in expenditure levels between deceased and non-deceased patients. For example, the difference in Total Medical Costs decreased from 0.759 before matching to 0.369 after matching, and the difference in Nursing Care Fees decreased from 1.121 to 0.490 after matching. Moreover, the estimated coefficients are significant at the 1% level. These results demonstrate that matching significantly reduced the differences in healthcare expenditures and cost variables between the two groups. This improvement enhances the reliability of the estimates and the comparability between groups, making causal inferences more robust and credible.

Table 3 presents the balance test conducted to assess the quality of Propensity Score Matching (PSM). Before matching, the balance coefficient (B) was 134.0, and the mean deviation was 15.2. After matching, the balance coefficient improved to B = 13.3, below the threshold of 25, and the ratio of variances (0.5 < R < 2) fell within the acceptable range. Additionally, the mean deviation was significantly reduced. These results indicate that PSM effectively minimized the distributional differences in explanatory variables between the deceased and non-deceased groups. Consequently, observable variable biases and estimation errors caused by sample self-selection were substantially mitigated, enhancing the reliability of the analysis. For robustness checks, this study also employed radius matching and kernel matching methods. The detailed results are provided in Supplementary Table 4. In summary, the PSM analysis revealed systematic differences in selection between the deceased and non-deceased groups, which were effectively reduced through the matching process. As a result, the conclusions drawn in this study reflect the true impact of mortality more reliably.

**Table 3:** Balance test results for matched variables after propensity score matching.

Sample	Ps R2	LR chi2	p > chi2	MeanBias	MedBias	B	R	%Var
Unmatched	0.189	2026.12	0.000	15.2	9.6	134.0*	1.46	67
Matched	0.003	10.16	1.000	1.8	1.3	13.3	1.03	100

* If B > 25%, R outside [0.5; 2].

Table 4 reports the regression results conducted on the matched samples, aimed at examining the impact of mortality on end-of-life healthcare expenditures among middle-aged and older patients with cardiovascular and cerebrovascular diseases. For the key variable, the mortality indicator exhibits positive and highly significant coefficients across all expenditure categories, indicating that mortality substantially increases various medical and caregiving costs.

**Table 4:** Regression analysis based on PSM-matched samples.

	(1)	(2)	(3)	(4)	(5)	(6)
	Total medical costs	Comprehensive service fees	Diagnosis fees	Treatment fees	Pharmaceutical fees	Nursing care fees
Death	0.343***	0.440***	0.357***	0.625***	0.499***	0.468***
	(15.032)	(19.659)	(11.312)	(7.913)	(17.882)	(14.504)
Control variables	Yes	Yes	Yes	Yes	Yes	Yes
Hospital fixed effects	Yes	Yes	Yes	Yes	Yes	Yes
City fixed effects	Yes	Yes	Yes	Yes	Yes	Yes
Disease fixed effects	Yes	Yes	Yes	Yes	Yes	Yes
Department fixed effects	Yes	Yes	Yes	Yes	Yes	Yes
Cons	10.175***	7.761***	8.540***	6.458***	8.740***	5.933***
	(61.591)	(46.797)	(41.425)	(12.811)	(44.701)	(31.733)
Adjusted *R*^2^	0.536	0.678	0.568	0.564	0.512	0.739
*N*	4037.000	3965.000	4037.000	4037.000	4037.000	4037.000

Total Medical Costs, Comprehensive Service Fees, and Diagnosis Fees increased by 34.3, 44.0, and 35.7%, respectively, with all increases statistically significant at the 1% level, reflecting considerable cost growth in these categories due to mortality. Treatment Fees, Pharmaceutical Fees, and Nursing Care Fees exhibited even more substantial increases of 62.5, 49.9, and 46.8%, respectively. The adjusted *R*^2^ values ranged from 0.512 to 0.739, indicating strong explanatory power of the models. These findings highlight the significant role of mortality in driving end-of-life healthcare expenditures across various cost categories.

## 4 Robustness check

### 4.1 Robustness check for propensity score matching

**Figure 1:**
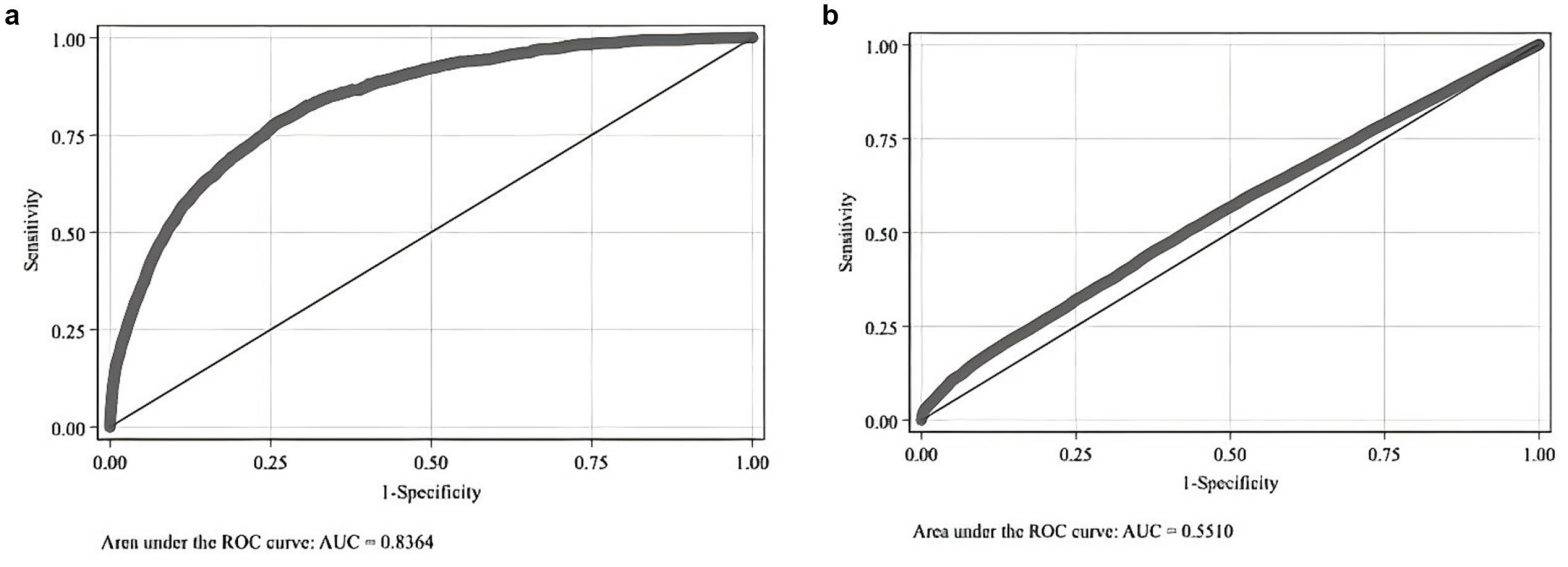
**(a)** Logit model for whether the patient was a middle-aged or older adult cardiovascular and cerebrovascular disease death case before matching. **(b)** Logit model for whether the patient was a middle-aged or older adult cardiovascular and cerebrovascular disease death case after matching.

Firstly, model accuracy is crucial for ensuring the effectiveness of the matching procedure. In this study, the Area Under the Curve (AUC) of the Receiver Operating Characteristic (ROC) curve is 0.8364, exceeding the threshold of 0.8, indicating that the model effectively distinguishes between deceased and non-deceased patients. This performance shows that the model’s predictive power for mortality is significantly better than random guessing, as shown in Figure 1a.

Secondly, tests for the Common Support Assumption and the Balancing Assumption were conducted after matching. The results of the Common Support test are displayed in Figure 1b. In Figure 1b, the AUC is 0.551, close to 0.5 and nearly parallel to the 45° line. This suggests that, post-matching, distinguishing between deceased and non-deceased middle-aged and older patients with cardiovascular and cerebrovascular diseases is challenging, thus satisfying the Common Support Assumption.

**Figure 2:**
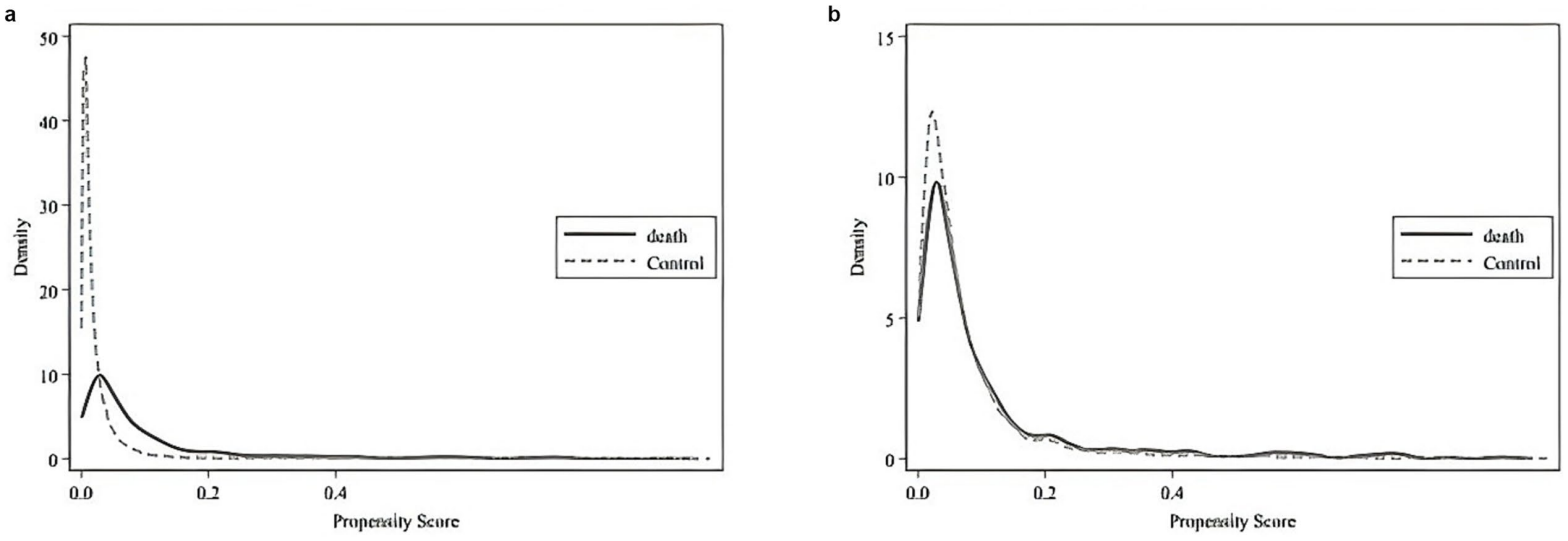
**(a)** Density function fit plot of the propensity score before matching. **(b)** Density function fit plot of the propensity score after matching.

Furthermore, comparing Figures 2a,b shows that, post-matching, the density function plots of the two groups are nearly identical. This similarity indicates that the characteristics of the two groups are well balanced after matching. The variable biases, *T*-tests, and LR tests reveal no significant differences between the variables of deceased and non-deceased patients, fulfilling the Balancing Assumption. Overall, the evaluation criteria confirm that the PSM matching process achieved satisfactory results.

### 4.2 Multidimensional fixed effects regression model

Table 5 presents the regression results from the Multidimensional Fixed Effects (MDFE) model. The findings indicate that Total Medical Costs, Comprehensive Service Fees, Diagnosis Fees, Treatment Fees, Pharmaceutical Fees, and Nursing Care Fees increased by 24.6, 36.4, 27.5, 36.2, 43.3, and 40.6%, respectively, all significant at the 1% level. These results confirm that deceased patients incur significantly higher expenditures than non-deceased patients across all medical cost categories. Notably, the growth rates of Treatment Fees, Pharmaceutical Fees, and Nursing Care Fees exhibit particularly high growth rates. The significantly higher hospitalization costs for deceased patients further support the robustness of the previous findings.

**Table 5:** Results of the multidimensional fixed effects regression model.

	(1)	(2)	(3)	(4)	(5)	(6)
	Total medical costs	Comprehensive service fees	Diagnosis fees	Treatment fees	Pharmaceutical fees	Nursing care fees
Death	0.246***	0.364***	0.275***	0.362***	0.433***	0.406***
	(13.207)	(19.312)	(9.869)	(4.584)	(17.510)	(16.068)
Control variables	Yes	Yes	Yes	Yes	Yes	Yes
Hospital fixed effects	Yes	Yes	Yes	Yes	Yes	Yes
City fixed effects	Yes	Yes	Yes	Yes	Yes	Yes
Disease fixed effects	Yes	Yes	Yes	Yes	Yes	Yes
Department fixed effects	Yes	Yes	Yes	Yes	Yes	Yes
Cons	9.587***	7.176***	7.888***	6.422***	7.818***	5.228***
	(244.295)	(180.602)	(134.193)	(38.515)	(149.859)	(98.065)
Adjusted *R*^2^	0.609	0.698	0.571	0.499	0.544	0.801
*N*	44349.000	43400.000	44349.000	44349.000	44349.000	44341.000

### 4.3 Difference-in-differences machine learning

Table 6 presents the regression results estimated using the Difference-in-Differences Machine Learning (DDML) model to evaluate the impact of mortality on various categories of medical expenditures. The analysis shows that the core variable, mortality, exerts a positive and highly significant effect across all categories of medical costs. Specifically, Total Medical Costs, Comprehensive Service Fees, Diagnosis Fees, Treatment Fees, Pharmaceutical Fees, and Nursing Care Fees increased by 46.3, 53.4, 55.4, 85.4, 73.6, and 69.9%, respectively. The increases in Treatment Fees, Pharmaceutical Fees, and Nursing Care Fees are particularly pronounced, highlighting the significant role of mortality in driving up expenditures in these categories. These findings further confirm the robustness of the previous analyses.

**Table 6:** Prediction results of the difference-in-differences machine learning model.

	(1)	(2)	(3)	(4)	(5)	(6)
	Total medical costs	Comprehensive service fees	Diagnosis fees	Treatment fees	Pharmaceutical fees	Nursing care fees
Death	0.463***	0.534***	0.554***	0.854***	0.736***	0.699***
	(21.294)	(19.250)	(16.783)	(9.813)	(29.972)	(16.245)
Hospital fixed effects	Yes	Yes	Yes	Yes	Yes	Yes
City fixed effects	Yes	Yes	Yes	Yes	Yes	Yes
Disease fixed effects	Yes	Yes	Yes	Yes	Yes	Yes
Department fixed effects	Yes	Yes	Yes	Yes	Yes	Yes
Cons	−0.000	−0.000	−0.000	−0.003	−0.000	−0.000
	(−0.105)	(−0.016)	(−0.048)	(−0.176)	(−0.038)	(−0.028)
*N*	44263.000	43316.000	44263.000	44263.000	44263.000	44263.000

Implemented via DDML with 4-fold cross-validation. The Y-model uses high-dimensional fixed effects regression (reghdfe v6.12), and the D-model uses logistic regression (logit v17.0). All hyperparameters follow Stata’s default settings with random seed fixed at 123.

## 5 Conclusion

The high costs of end-of-life (EOL) medical care impose significant pressure on social health insurance systems, posing a major challenge in healthcare management. This study employs Propensity Score Matching (PSM) to analyze inpatient discharge summary data from H Province, China (2018–2019), examining the relationship between mortality and EOL medical expenditures among middle-aged and older adult patients with cardiovascular and cerebrovascular diseases. Robustness checks are conducted using Multidimensional Fixed Effects (MDFE) and Difference-in-Differences Machine Learning (DDML) models. The findings indicate that healthcare expenditures for deceased patients are significantly higher than those at other life stages, confirming the existence of the “nearing-death effect.” The Increases in treatment, pharmaceutical, and nursing care fees are particularly pronounced.

Global evidence aligns with this study’s findings, confirming a consistent trend of rising EOL medical expenditures across diverse healthcare systems. For instance, studies in the United States report that end-of-life cancer care is associated with the highest costs, with expenditures 2.52 times and 19.9 times higher in the final stage compared to the initial and ongoing treatment phases, respectively (14). Similarly, research in Singapore finds that EOL medical costs rise significantly, accounting for 61% of total death-related expenses in the 12 months preceding death and increasing to 94% in the final month (15). Consistently, data from New Zealand indicate that per capita medical expenses in the 6 months before death are 10 times the average annual level (16). These consistent findings further substantiate this study’s conclusion that healthcare expenditures for deceased patients are significantly higher than those at other life stages, providing robust evidence for the existence of the “nearing-death effect.”

This shared pattern of escalating EOL medical costs not only underscores the urgent need for comprehensive measures to safeguard the economic security and dignity of the older adult in aging societies. It also highlights the global demand for innovative end-of-life care systems, optimized health insurance payment mechanisms, and the integrated cross-lifecycle health management frameworks. Based on empirical findings and global experiences, this paper proposes four policy recommendations.

Firstly, Innovating the end-of-life (EOL) care system requires the establishment of a comprehensive and well-integrated service network. A structured, multi-tiered EOL care model should be implemented, comprising a three-level palliative care framework: specialized wards in tertiary hospitals for critical cases, hospice centers in secondary hospitals for transitional care, and community-based home services for non-intensive support. This structured approach facilitates the seamless integration of healthcare resources across different levels, enhancing continuity and efficiency in EOL care delivery. At the same time, strengthening multidisciplinary teams and integrating medical and caregiving resources are essential to delivering holistic and patient-centered EOL care. Developing a digitalized EOL care platform will enable a full-service continuum, ranging from “acute symptom control” to “symptom management” and “long-term care.” This initiative will improve the accessibility and patient-centeredness of EOL care, ensuring that services are both effective and compassionate.

Secondly, reform healthcare payment mechanisms and build a multi-tiered financial protection system. Anchored in Value-Based Payment Design (VBPD) reforms, this strategy includes increasing the DRG payment weighting for end-of-life care, incentivizing medical institutions to optimize resource allocation for cardiovascular and cerebrovascular disease management in middle-aged and older adult populations while enhancing the quality of terminal care (17).

Additionally, expanding insurance coverage to incorporate home-based hospice care and remote monitoring services can provide cost-effective alternatives to inpatient care. At the same time, introducing an expenditure cap system will help mitigate the risk of catastrophic household medical expenses, ensuring financial protection for families facing the high costs of terminal illness. Furthermore, the Long-Term Care Insurance (LTCI) pilot programs should be expanded by developing dynamically adaptive disability assessment models and actuarial indices to enhance chronic disease management for aging populations with cardiovascular and cerebrovascular conditions (18). Strengthening long-term care financing mechanisms will ensure the sustainability of EOL care services, particularly for those requiring prolonged support. This approach aims to balance the rational allocation of medical resources with the financial burdens on patients (19).

Thirdly, establish a cross-lifecycle health management model to alleviate healthcare pressures. Building on the concept of “prevention first,” develop a health management ecosystem that strengthens early intervention through initiatives such as the Cardiovascular and Cerebrovascular Health Passport program and Chronic Disease Management Quality Payment (QBP) schemes. Integrate data from electronic medical records, wearable devices, and other sources to establish a health data bank that can predict the medical needs of the middle-aged and older adult population (20). Innovate social support models by mobilizing social capital to participate in the construction of community-based end-of-life care facilities. This will create a comprehensive “Prevention—Early Warning—Protection” health management system that addresses the root causes of healthcare pressure in an aging society.

Fourthly, pilot initiatives and implement end-of-life care protection mechanisms. Legislate to clearly define patients’ rights to end-of-life care, and establish a monitoring system that includes indicators such as cost control rates and quality of life indices. Launch policy pilots in aging pioneer regions such as the Yangtze River Delta and Chengdu-Chongqing areas (21), overcoming current limitations in the medical insurance catalog and pricing. These pilots will provide valuable insights for nationwide implementation, ensuring the scientific and sustainable execution of the reform plan.

## 6 Limitations

Despite offering valuable insights into the end-of-life (EOL) medical expenditures for middle-aged and older adult patients with cardiovascular and cerebrovascular diseases, this study has several limitations. First, the data used in this study was sourced from the 2018–2019 medical insurance settlement platform in H Province, China. While the conclusions may have some external validity for other regions and healthcare systems, there are inherent limitations. Specifically, the study did not include out-of-pocket expenses, informal medical costs, or non-insured services, which may lead to an underestimation of EOL medical expenditures. Additionally, the study does not account for long-term trends in medical expenses or the potential impacts of significant public health events such as the COVID-19 pandemic.

Second, although the study identifies a significant “nearing-death effect,” it did not thoroughly investigate the objective factors driving the increased EOL expenditures, such as the role of patients’ and families’ subjective treatment preferences, or physicians’ decision-making. Further research could explore whether these increases in expenditures are attributable to medical necessity or patient/family preferences.

Moreover, this study did not assess whether the increase in EOL medical expenditures resulted in improved quality of life or prolonged survival for the patients, which would be crucial for evaluating the effective use of medical resources. Finally, the study did not delve into the influence of policy factors such as health insurance reforms, hospital pricing mechanisms, or drug price adjustments, all of which could significantly impact the pattern of terminal medical expenditures.

Future research could address these limitations by expanding the scope, incorporating additional individual characteristics, assessing the impact of medical expenditures on patient well-being, and analyzing the role of healthcare policies in shaping expenditure patterns.

## Data Availability

The data analyzed in this study is subject to the following licenses/restrictions: the data used in this study are administrative in nature and have been utilised in compliance with the local regulatory and legal frameworks governing the use of administrative data. The datasets generated and analyzed during the study are not publicly available due to data confidentiality but can be obtained from the corresponding author upon reasonable request. Requests to access these datasets should be directed to https://ybj.hubei.gov.cn/.

## References

[1] Emanuel EJ Emanuel LL. The economics of dying. The illusion of cost savings at the end of life. N Engl J Med. (1994) 330:540–4. doi: 10.1056/NEJM199402243300806, PMID: 8302321

[2] Aldridge MD Kelley AS. The myth regarding the high cost of end-of-life care. Am J Public Health. (2015) 105:2411–5. doi: 10.2105/AJPH.2015.302889, PMID: 26469646 PMC4638261

[3] Li L Hu L Ji J Mckendrick K Moreno J Kelley AS. Determinants of Total end-of-life health care costs of Medicare beneficiaries: a quantile regression forests analysis. J Gerontol A Biol Sci Med Sci. (2022) 77:1065–71. doi: 10.1093/gerona/glab176, PMID: 34153101 PMC9071433

[4] Stahmeyer JT Hamp S Zeidler J Eberhard S. Gesundheitsausgaben und die Rolle des Alters: Eine detaillierte Analyse der Kosten von Überlebenden und Verstorbenen [Healthcare expenditure and the impact of age: a detailed analysis for survivors and decedents. Bundesgesundheitsblatt Gesundheitsforschung Gesundheitsschutz. (2021) 64:1307–14. doi: 10.1007/s00103-021-03385-y, PMID: 34258630

[5] Schouela N Kyeremanteng K Thompson LH Neilipovitz D Shamy M D'Egidio G. Cost of futile ICU Care in one Ontario Hospital. Inquiry. (2021) 58:469580211028577. doi: 10.1177/00469580211028577, PMID: 34218711 PMC8261843

[6] Cutler D Skinner JS Stern AD Wennberg D. Physician beliefs and patient preferences: a new look at regional variation in health care Spendingf. Am Econ J Econ Policy. (2019) 11:192–221. doi: 10.1257/pol.20150421, PMID: 32843911 PMC7444804

[7] Reinharz D Lesage AD Contandriopoulos AP. Cost-effectiveness analysis of psychiatric deinstitutionalization. Can J Psychiatr. (2000) 45:533–8. doi: 10.1177/070674370004500603, PMID: 10986570

[8] Baily MA. Futility, autonomy, and cost in end-of-life care. J Law Med Ethics. (2011) 39:172–82. doi: 10.1111/j.1748-720X.2011.00586.x, PMID: 21561512

[9] Hughes SL Weaver FM Giobbie-Hurder A Manheim L Henderson W Kubal JD. Effectiveness of team-managed home-based primary care: a randomized multicenter trial. JAMA. (2000) 284:2877–85. doi: 10.1001/jama.284.22.2877, PMID: 11147984

[10] Vilpert S Borrat-Besson C Domenico Borasio G Maurer J. Correction: associations of end-of-life preferences and trust in institutions with public support for assisted suicide: evidence from nationally representative survey data of older adults in Switzerland. PLoS One. (2020) 15:e0234954. doi: 10.1371/journal.pone.0234954, PMID: 32555749 PMC7302435

[11] Pennington M Baker R Brouwer W Mason H Hansen DG Robinson A. Comparing WTP values of different types of QALY gain elicited from the general public. Health Econ. (2015) 24:280–93. doi: 10.1002/hec.3018, PMID: 25625510

[12] Da Y Fu H Li L. The impact of changes in patient cost-sharing on medical expenditures and health outcomes: empirical evidence from hospitalisation medical record data. China Econ Q. (2020) 19:1441–66. doi: 10.13821/j.cnki.ceq

[13] Zang W Chen C Zhao S. Social health insurance, disease heterogeneity, and medical expenditures. Econ Res J. (2020) 55:64–79. Available at: https://kns.cnki.net/kcms2/article/abstract?v=fNwONIwGMRJTyZIJ2mTZXhh2fA-C7OcGdGQjE3qotTY8TQkRMkVMRo5e45r1b_pzx5PX-Nt1NiKrnrcRGVFdIh1iYoAfRT4wf1Ve87nK71HzFrMK8jRcxkmaS4LoXUlrDRyle8JXj-myVazG8joSARLnYvUt28fEMt5V_8uOgqFFyB49ZJqLP7xm2_j7kPLB_sWDXlqevas=&uniplatform=NZKPT&language=CHS

[14] Mariotto AB Enewold L Zhao J Zeruto CA Yabroff KR. Medical care costs associated with Cancer survivorship in the United States. Cancer Epidemiol Biomarkers Prev. (2020) 29:1304–12. doi: 10.1158/1055-9965.EPI-19-1534, PMID: 32522832 PMC9514601

[15] Kaur P Wu HY Hum A Heng BH Tan WS. Medical cost of advanced illnesses in the last-year of life-retrospective database study. Age Ageing. (2022) 51:afab212. doi: 10.1093/ageing/afab212, PMID: 34673931

[16] Blakely T Atkinson J Kvizhinadze G Nghiem N McLeod H Wilson N. Health system costs by sex, age and proximity to death, and implications for estimation of future expenditure. N Z Med J. (2014) 127:12–25. 24816953

[17] Quinn KL Hsu AT Meaney C Qureshi D Tanuseputro P Seow H. Association between high cost user status and end-of-life care in hospitalized patients: a national cohort study of patients who die in hospital. Palliat Med. (2021) 35:1671–81. doi: 10.1177/02692163211002045, PMID: 33781119 PMC8532234

[18] Ma H Jia E Ma H Pan Y Jiang S Xiong J. Preferences for public long-term care insurance among middle-aged and elderly residents: a discrete choice experiment in Hubei Province, China. Front Public Health. (2023) 11:1050407. doi: 10.3389/fpubh.2023.1050407, PMID: 36778541 PMC9909219

[19] Hiriscau EI Buzdugan EC Hui LA Bodolea C. Exploring the relationship between frailty, functional status, polypharmacy, and quality of life in elderly and middle-aged patients with cardiovascular diseases: a one-year follow-up study. Int J Environ Res Public Health. (2022) 19:2286. doi: 10.3390/ijerph19042286, PMID: 35206472 PMC8871852

[20] Kim YS Lee J Moon Y Kim KJ Lee K Choi J. Unmet healthcare needs of elderly people in Korea. BMC Geriatr. (2018) 18:98. doi: 10.1186/s12877-018-0786-3, PMID: 29678164 PMC5910628

[21] Fan P Li H Xu H Rong C. Impact of medical-nursing combined policy pilot on hospitalization frequency of middle-aged and older patients with chronic diseases: a quasi-experimental study based on China health and retirement longitudinal study. Front Public Health. (2024) 12:1450828. doi: 10.3389/fpubh.2024.1450828, PMID: 39463894 PMC11502400

