# Supplementary material for: Dying to pay: end-of-life medical costs for middle-aged and older adult patients with cardiovascular and cerebrovascular diseases
Source: Front Public Health. 2025 Mar 12;13:1548999. doi: 10.3389/fpubh.2025.1548999 (PMC11949274; doi:10.3389/fpubh.2025.1548999)
Supplement: Supplementary file 1 [file Table_1.docx]

| **TABLE1** Research object ICD-10 coding | | | | |  |
| --- | --- | --- | --- | --- | --- |
| **Variable type** | **The name of the variable** | **Variable level** |  | | **ICD-10 coding** |
| Disease Attributed | Type of disease | Valvular heart disease | diseA | I05. 0、I05. 1、I05. 2、I05. 8、I05. 9、I06.0、I06.1、I06. 2、I06. 8、I06.9、I07. 0、I07. 1、I07. 2、I07.8、I07. 9、.I08. 0、I08. 1、I08. 2、I08. 3、I08. 8、I08. 9、I34. 0、I34. 1、I34. 2、I34. 8、I34. 9、I35. 0、I35. 1、I35. 2、I35. 3、.I35. 8、I35. 9、I36. 0、I36. 1、I36. 2、I36. 8、I36. 9、I37. 0、I37. 1、I37. 2、I37. 8、I37. 9 | |
|  |  | Rheumatic heart disease | diseB | I09. 0、I09. 1、I09. 2、I09. 8、I09. 9 | |
|  |  | Hypertension | diseC | I10、I10. 0、I10. 1、I10. 2、I10. 3、I10. 4、I10. 5、I10. 6、I10. 8、I10. 9 | |
|  |  | Hypertensive Heart Disease or Kidney Disease | diseD | I11. 0、I11. 1、I11. 9、I12. 0、I12. 9、I13. 0、I13. 1、I13. 2、I13. 9 | |
|  |  | Secondary hypertension | diseE | I15. 0、I15. 1、I15. 2、I15. 8、I15. 9 | |
|  |  | Angina | diseF | I20. 0、I20. 1、I20. 8、I20. 9 | |
|  |  | Myocardial Infarction | diseG | I21. 0、I21. 1、I21. 2、I21. 3、I21. 4、I21. 9、I22. 0、I22. 1、I22. 8、I22. 9、I23. 0、I23. 1、I23. 2、I23. 3、I23. 4、 I23. 5、I23. 6、I23. 8 | |
|  |  | Coronary Heart Disease | diseH | I25. 1 | |
|  |  | Ischemic Heart Disease | diseI | I24. 0、I24. 1、I24. 8、I24. 9、I25. 0、I25. 2、I25. 3、I25. 4、I25. 5、I25. 6、I25. 8、I25. 9 | |
|  |  | Cor pulmonale | diseJ | I26. 0、I26. 9、I27. 0、I27. 1、I27. 2、I27. 8、I27. 9、I28. 0、I28. 1、I28. 8、I28. 9 | |
|  |  | Other types of heart disease | diseK | I30. 0、I30. 1、I30. 8、I31. 2、I31. 8、I32. 1、I32. 8、I33. 9、I39. 0、I39. 1、I39. 2、I39. 3、I39. 4、I39. 8、I40. 1、 I41. 0、I41. 1、I41. 2、I41. 8、I43. 0、I43. 1、I43. 2、I48. 2、I483、I48. 4、I48. 9、I51. 0、 I51. 1、I51. 2、 I51. 6、 I51. 8、I51. 9、I52. 0、I52. 1、I52. 8 | |
|  |  | Cardiomyopathy and myocarditis | diseL | I30. 9、I31. 0、I31. 1、I31. 3、I31. 9、I33. 0、I38. 0、I40. 0、I40. 8、I40. 9、I42. 0、I42. 1、I42. 2、I42. 3、I42. 4、 I42. 5、I42. 7、I42. 8、I42. 9 | |
|  |  | Heart Block | diseM | I44. 0、I44. 1、I44. 2、I44. 3、I44. 4、I44. 5、I44. 6、I44. 7、I45. 0、I45. 1、I45. 2、I45. 3、I45. 4、I45. 5、I45. 6、 I45. 7、I45. 8、I45. 9 | |
|  |  | Cardiac rhythm disturbances and atrial fibrillation treated | diseN | I47. 0、I47. 1、I47. 2、I47. 9、I48. 0、I48. 1、I49. 1、I49. 2、I49. 3、I49. 4、I49. 5、I49. 8、I49. 9 | |
|  |  | Heart Failure | diseO | I50. 0、I50. 1、I50. 4、I50. 5、I50. 6、I50. 9 | |
|  |  | Cardiac Hypertrophy | diseP | I51. 7 | |
|  |  | Cerebral infarction or Intracerebral Hemorrhage | diseQ | I60. 0、I60. 1、I60. 2、I60. 3、I60. 4、I60. 5、I60. 6、I60. 7、I60. 8、I60. 9、I61. 0、I61. 1、I61. 2、I61. 3、I61. 4、 I61. 5、I61. 6、I61. 7、I61. 8、I61. 9、I62. 0、I62. 1、I62. 8、I62. 9、I63. 0、I63. 1、I63. 2、I63. 3、I63. 4、I63. 5、 I63. 6、I63. 7、I63. 8、I63. 9、I64. 0、I69. 0、I69. 1、I69. 2、I69. 3、I69. 4、I69. 8 | |
|  |  | Cerebrovascular Stenosis | diseR | I65. 0、I65. 1、I65. 2、I65. 3、I65. 8、I65. 9、I66. 0、I66. 1、I66. 2、I66. 3、I66. 4、I66. 8、I66. 9 | |
|  |  | Arteriosclerotic atrophy of the brain/Atherosclerosis of cerebral artery | diseS | I67. 2 | |
|  |  | The Other of Cerebral Vascular Disease | diseT | I67. 3、I67. 5、I67. 6、I67. 7、I67. 8、I67. 9 | |
|  |  | Arterial, arteriole, and capillary disease | diseU | I70. 0、I70. 1、I70. 2、I70. 3、I70. 8、I70. 9、I71. 0、I71. 1、I71. 2、I71. 3、I71. 4、I71. 5、I71. 6、I71. 8、I71. 9、 I72. 0、I72. 1、I72. 2、I72. 3、I72. 4、I72. 5、I72. 6、I72. 8、I72. 9、I73. 0、I73. 1、I73. 2、I73. 8、I73. 9、I74. 0、 I74. 1、I74. 2、I74. 3、I74. 4、I74. 5、I74. 8、I74. 9、I77. 0、I77. 1、I77. 2、I77. 3、I77. 4、I77. 5、I77. 6、I77. 8、 I77. 9、I78. 0、I78. 1、I78. 8、I78. 9、I79. 0、I79. 1 | |
|  |  | Venous, lymphatic and lymph node disease | diseV | I80. 0、I80. 1、I80. 2、I80. 3、I80. 9、I81. 0、I81. 2、I82. 0、I82. 1、I82. 2、I82. 3、I82. 8、I82. 9、I83. 0、I83. 1、 I83. 2、I83. 3、I83. 8、I83. 9、I85. 0、I85. 9、I86. 0、I86. 1、I86. 2、I86. 3、I86. 4、I86. 8、I87. 0、I87. 1、I87. 2、 I87. 8、I87. 9、I88. 0、I88. 1、I88. 8、I88. 9、I89. 0、I89. 1、I89. 8、I89. 9 | |
|  |  | Other diseases of the circulatory system | diseW | I00. 0、I01. 0、I01. 1、I01. 2、I01. 8、I01. 9、I02. 0、I02. 9、I46. 0、I46. 1、I46. 2、I46. 9、I49. 0、I95. 0、I95. 1、 I95. 2、I95. 8、I95. 9、I97. 0、I97. 1、I97. 2、I97. 8、I97. 9、I98. 0、I98. 1、I98. 3、I99. 0、I99. 1、I99. 2 | |
|  | Complications | Type 2 diabetes mellitus | campA | O241、E11、E110、E111、E112、E113、E114、E115、E116、E117、E118、E119 | |
|  |  | Hyperlipidemia | campB | K861、0992、E780、E781、E782、E783、E784、E785、E898 | |
|  |  | Fatty liver disease | campC | K700、K760、O266 | |

**TABLE2** Descriptive Statistical Analysis of Diseases

| **Variables** | **Full Sample** | | **Control Group** | **Experimental Group** |  |
| --- | --- | --- | --- | --- | --- |
|  | **Mean** | **Standard Deviation** | **Mean** | **Mean** | **MeanDiff** |
| diseA | 0.039 | 0.194 | 0.039 | 0.044 | -0.005 |
| diseB | 0.004 | 0.065 | 0.004 | 0.004 | -0.000 |
| diseC | 0.638 | 0.481 | 0.642 | 0.505 | 0.137*** |
| diseD | 0.028 | 0.166 | 0.028 | 0.037 | -0.009* |
| diseE | 0.013 | 0.112 | 0.013 | 0.010 | 0.002 |
| diseF | 0.030 | 0.171 | 0.030 | 0.024 | 0.006 |
| diseG | 0.016 | 0.125 | 0.015 | 0.061 | -0.046*** |
| diseH | 0.204 | 0.403 | 0.203 | 0.261 | -0.058*** |
| diseI | 0.050 | 0.218 | 0.049 | 0.073 | -0.023*** |
| diseJ | 0.038 | 0.192 | 0.037 | 0.085 | -0.048*** |
| diseK | 0.027 | 0.163 | 0.027 | 0.043 | -0.016*** |
| diseL | 0.010 | 0.100 | 0.010 | 0.016 | -0.006* |
| diseM | 0.017 | 0.130 | 0.017 | 0.016 | 0.001 |
| diseN | 0.062 | 0.240 | 0.061 | 0.094 | -0.034*** |
| diseO | 0.154 | 0.361 | 0.149 | 0.348 | -0.199*** |
| diseP | 0.010 | 0.100 | 0.010 | 0.010 | 0.001 |
| diseQ | 0.392 | 0.488 | 0.392 | 0.394 | -0.002 |
| diseR | 0.053 | 0.224 | 0.054 | 0.022 | 0.031*** |
| diseS | 0.025 | 0.155 | 0.025 | 0.012 | 0.013*** |
| diseT | 0.012 | 0.109 | 0.012 | 0.003 | 0.009*** |
| diseU | 0.110 | 0.312 | 0.110 | 0.084 | 0.026*** |
| diseV | 0.073 | 0.261 | 0.073 | 0.082 | -0.009 |
| diseW | 0.008 | 0.086 | 0.005 | 0.099 | -0.094*** |
| N | 44341 | 44341 | 43185 | 1156 |  |

Note: The values in parentheses represent t-values; ***, **, and * denote significance at the 1%, 5%, and 10% levels, respectively. The same applies to the following tables.

**TABLE3** Disease Cost Proportion and Scale

|  | **Data Scale** | **Total Medical Costs** | **Comprehensive Service Fees** | **Diagnosis Fees** | **Treatment Fees** | **Pharmaceutical Fees** | **Nursing Care Fees** |
| --- | --- | --- | --- | --- | --- | --- | --- |
| diseA | .03908347 | 10.663644 | 8.5728548 | 8.8188682 | 7.3552038 | 9.2513938 | 6.2492876 |
| diseB | .00428497 | 10.248618 | 8.4514539 | 8.4348556 | 6.5550659 | 8.7993595 | 6.4364998 |
| diseC | .63814528 | 10.205005 | 8.1955296 | 8.2434535 | 6.646244 | 8.6904872 | 6.1445299 |
| diseD | .02841614 | 10.18454 | 8.2758262 | 8.4617937 | 6.7370383 | 8.7962613 | 6.2697699 |
| diseE | .01262939 | 9.9714556 | 7.9803781 | 7.7402911 | 6.3528201 | 8.4534629 | 5.5375469 |
| diseF | .02999481 | 10.08899 | 8.0165852 | 8.2683869 | 6.1755715 | 8.6057909 | 6.051239 |
| diseG | .0158544 | 10.838352 | 8.696363 | 9.0094267 | 7.9364324 | 9.2960683 | 6.7874389 |
| diseH | .20437067 | 10.188399 | 8.2118401 | 8.3279637 | 6.0899227 | 8.7460237 | 6.1336595 |
| diseI | .04986356 | 10.207401 | 8.2602115 | 8.214261 | 6.244377 | 8.7909752 | 6.2791176 |
| diseJ | .03829413 | 10.376114 | 8.6239334 | 8.5226604 | 6.1859985 | 9.0807383 | 6.3800976 |
| diseK | .02728851 | 10.436335 | 8.3720402 | 8.5830944 | 6.7647445 | 9.069348 | 6.1683578 |
| diseL | .01019373 | 10.461197 | 8.5699818 | 8.7112032 | 6.7333691 | 9.1715564 | 6.5383638 |
| diseM | .01720755 | 10.368089 | 8.2944962 | 8.5386126 | 7.1656696 | 8.7515664 | 6.2639884 |
| diseN | .06150064 | 10.291217 | 8.2455797 | 8.4374523 | 6.5609861 | 8.8216694 | 6.2961897 |
| diseO | .15380799 | 10.326984 | 8.4657483 | 8.5441597 | 6.3254423 | 8.9765872 | 6.4512037 |
| diseP | .01010352 | 10.484132 | 8.4552889 | 8.7371358 | 6.9342464 | 9.0471892 | 6.2651382 |
| diseQ | .39198484 | 10.332656 | 8.3486953 | 8.3813665 | 6.654839 | 8.8876557 | 6.2274205 |
| diseR | .05308856 | 10.16989 | 8.0044236 | 8.4121752 | 5.9022212 | 8.7959645 | 5.6622489 |
| diseS | .02467243 | 10.030416 | 7.8209078 | 8.3159382 | 5.1571488 | 8.6941496 | 5.2661582 |
| diseT | .01213324 | 10.139966 | 8.2404476 | 8.3039403 | 5.9963963 | 8.5791916 | 6.1949274 |
| diseU | .10953745 | 10.351312 | 8.2230879 | 8.5366073 | 6.8050828 | 8.9254007 | 6.0731833 |
| diseV | .07336325 | 10.645426 | 8.5482358 | 8.6206324 | 7.7069905 | 9.159367 | 6.5650418 |
| diseW | .00750998 | 10.925039 | 9.0093925 | 8.8900518 | 8.1446295 | 9.5261735 | 7.1401715 |
| campA | .1910647 | 10.188414 | 8.1753976 | 8.3615332 | 6.4515639 | 8.7515452 | 6.065892 |
| campB | .07180713 | 9.89319 | 7.768185 | 8.2435844 | 5.5483128 | 8.4514616 | 5.6335347 |
| campC | .0321824 | 9.9773784 | 7.8185654 | 8.3690979 | 5.9247911 | 8.4987322 | 5.6463845 |

**Table 4** Radius Matching and Kernel Matching

| Variable | Sample | Treated | Controls | Difference | S.E. | T-stat |
| --- | --- | --- | --- | --- | --- | --- |
| Radius Matching |  |  |  |  |  |  |
| Total Medical Costs | Unmatched | 11.027 | 10.268 | 0.759 | 0.028 | 27.22 |
|  | ATT | 11.024 | 10.630 | 0.393 | 0.024 | 16.46 |
| Comprehensive Service Fees | Unmatched | 9.161 | 8.212 | 0.949 | 0.032 | 29.24 |
|  | ATT | 9.156 | 8.714 | 0.442 | 0.029 | 15.03 |
| Diagnosis Fees | Unmatched | 9.031 | 8.359 | 0.672 | 0.035 | 19.18 |
|  | ATT | 9.028 | 8.627 | 0.401 | 0.032 | 12.51 |
| Treatment Fees | Unmatched | 8.121 | 6.794 | 1.327 | 0.105 | 12.58 |
|  | ATT | 8.116 | 7.289 | 0.827 | 0.092 | 9.02 |
| Pharmaceutical Fees | Unmatched | 9.812 | 8.763 | 1.049 | 0.034 | 30.71 |
|  | ATT | 9.808 | 9.182 | 0.626 | 0.027 | 23.24 |
| Nursing Care Fees | Unmatched | 7.402 | 6.282 | 1.121 | 0.047 | 23.71 |
|  | ATT | 7.396 | 6.859 | 0.537 | 0.042 | 12.77 |
| Kernel Matching |  |  |  |  |  |  |
| Total Medical Costs | Unmatched | 11.027 | 10.268 | 0.759 | 0.028 | 27.22 |
|  | ATT | 11.026 | 10.540 | 0.487 | 0.024 | 20.40 |
| Comprehensive Service Fees | Unmatched | 9.161 | 8.212 | 0.949 | 0.032 | 29.24 |
|  | ATT | 9.160 | 8.592 | 0.568 | 0.029 | 19.38 |
| Diagnosis Fees | Unmatched | 9.031 | 8.359 | 0.672 | 0.035 | 19.18 |
|  | ATT | 9.030 | 8.570 | 0.460 | 0.032 | 14.40 |
| Treatment Fees | Unmatched | 8.121 | 6.794 | 1.327 | 0.105 | 12.58 |
|  | ATT | 8.119 | 7.177 | 0.942 | 0.092 | 10.28 |
| Pharmaceutical Fees | Unmatched | 9.812 | 8.763 | 1.049 | 0.034 | 30.71 |
|  | ATT | 9.812 | 9.078 | 0.734 | 0.027 | 27.26 |
| Nursing Care Fees | Unmatched | 7.402 | 6.282 | 1.121 | 0.047 | 23.71 |
| Total Medical Costs | ATT | 7.401 | 6.719 | 0.682 | 0.042 | 16.30 |
